# Correction: Evaluation of antigen-detecting and antibody-detecting diagnostic test combinations for diagnosing melioidosis

**DOI:** 10.1371/journal.pntd.0010095

**Published:** 2022-06-02

**Authors:** The PLOS Neglected Tropical Diseases Staff

S2 Table contains errors in the ‘Cases’ and ‘Sensitivity’ columns, including the ‘No. of cases with positive results’, the ‘No. of cases with negative results’, and the ‘No. of cases with positive results/N (in the parentheses)’. The correct S2 Table can be viewed below.

S3 Table contains two wrong percentage point calculations for the values ‘(41/87)’ and ‘(56/99)’. The correct S3 Table can be viewed below.

S4 Table contains three wrong percentage point calculations for the values ‘(162/169)’, ‘(167/169)’, and ‘(122/123)’. The correct S4 Table can be viewed below.

## Supporting information

S2 Table Sensitivity and specificity of a combination between CPS-LFI and Hcp1-ELISA and a combination of CPS-LFI and OPS-ELISA using different OD cut-off values. This table includes supplementary data.
(DOCX)

S3 Table Diagnostic test results in different groups of melioidosis case patients. This table includes supplementary data.
(DOCX)

S4 Table Diagnostic test results in different groups of control patients. This table includes supplementary data.
(DOCX)
