# Supplementary material for: Evaluation of antigen-detecting and antibody-detecting diagnostic test combinations for diagnosing melioidosis
Source: PLoS Negl Trop Dis. 2021 Nov 2;15(11):e0009840. doi: 10.1371/journal.pntd.0009840 (PMC8562799; doi:10.1371/journal.pntd.0009840)
Supplement: S1 Table — (DOCX) [file pntd.0009840.s001.docx]

**S1 Table. Sensitivity and specificity of Hcp1-ELISA and OPS-ELISA using different OD cut-off values**

| Assay | OD cut-off  (of ELISA) | Cases (N = 192) | | Sensitivity | Controls (N = 502) | | | Specificity | |
| --- | --- | --- | --- | --- | --- | --- | --- | --- | --- |
|  |  | No. of cases with positive results | No. of cases negative results | (No. of cases with positive results/N) | No. of controls with positive results | | No. of controls with negative results | (No. of controls with negative results/N) | |
| Hcp1-ELISA | 2.721 | 105 | 87 | 54.7% (105/192) | 26 | 476 | | 94.8% (476/502) |  |
|  | 2.758* | 103 | 89 | 53.6% (103/192) | 25 | 477 | | 95.0% (477/502) |  |
|  | 2.797 | 100 | 92 | 52.1% (100/192) | 23 | 479 | | 95.4% (479/502) |  |
|  | 2.824 | 99 | 93 | 51.6% (99/192) | 21 | 481 | | 95.8% (481/502) |  |
|  | 2.832 | 99 | 93 | 51.6% (99/192) | 20 | 482 | | 96.0% (482/502) |  |
|  | 2.896 | 97 | 95 | 50.5% (97/192) | 19 | 483 | | 96.2% (483/502) |  |
|  | 2.912 | 97 | 95 | 50.5% (97/192) | 18 | 484 | | 96.4% (484/502) |  |
|  | 2.931 | 96 | 96 | 50.0% (96/192) | 17 | 485 | | 96.6% (485/502) |  |
| OPS-ELISA | 2.822 | 93 | 99 | 48.4% (93/192) | 27 | 475 | | 94.6% (475/502) |  |
|  | 2.839* | 93 | 99 | 48.4% (93/192) | 25 | 477 | | 95.0% (477/502) |  |
|  | 2.841 | 93 | 99 | 48.4% (93/192) | 24 | 478 | | 95.2% (478/502) |  |
|  | 2.844 | 93 | 99 | 48.4% (93/192) | 23 | 479 | | 95.4% (479/502) |  |
|  | 2.861 | 93 | 99 | 48.4% (93/192) | 22 | 480 | | 95.6% (480/502) |  |
|  | 2.865 | 93 | 99 | 48.4% (93/192) | 21 | 481 | | 95.8% (481/502) |  |
|  | 3.065 | 81 | 111 | 42.2% (81/192) | 20 | 482 | | 96.0% (482/502) |  |
|  | 3.077 | 79 | 113 | 41.1% (79/192) | 19 | 483 | | 96.2% (483/502) |  |
|  | 3.100 | 79 | 113 | 41.1% (79/192) | 18 | 484 | | 96.4% (484/502) |  |
|  | 3.189 | 76 | 116 | 39.6% (76/192) | 17 | 485 | | 96.6% (485/502) |  |

***** The lowest OD cut-offs that gave a specificity of the ELISA at 95%.
