# Supplementary material for: Evaluation of antigen-detecting and antibody-detecting diagnostic test combinations for diagnosing melioidosis
Source: PLoS Negl Trop Dis. 2021 Nov 2;15(11):e0009840. doi: 10.1371/journal.pntd.0009840 (PMC8562799; doi:10.1371/journal.pntd.0009840)
Supplement: S2 Table — (DOCX) [file pntd.0009840.s002.docx]

**S2 Table. Sensitivity and specificity of a combination between CPS-LFI and Hcp1-ELISA and a combination of CPS-LFI and OPS-ELISA using different OD cut-off values**

| Assay | OD cut-off  (of ELISA) | Cases (N = 192) | | Sensitivity | Controls (N = 502) | | Specificity |
| --- | --- | --- | --- | --- | --- | --- | --- |
|  |  | No. of cases with positive results | No. of cases negative results | (No. of cases with positive results/N) | No. of controls with positive results | No. of controls with negative results | (No. of controls with negative results/N) |
| CPS-LFI and | 2.721 | 105 | 87 | 71.4% (105/192) | 33 | 469 | 93.4% (469/502) |
| Hcp1-ELISA | 2.758 | 103 | 89 | 70.8% (103/192) | 32 | 470 | 93.6% (470/502) |
|  | 2.797 | 100 | 92 | 69.3% (100/192) | 30 | 472 | 94.0% (472/502) |
|  | 2.824 | 99 | 93 | 68.8% (99/192) | 28 | 474 | 94.4% (474/502) |
|  | 2.832 | 99 | 93 | 68.8% (99/192) | 27 | 475 | 94.6% (475/502) |
|  | 2.896 | 97 | 95 | 67.7% (97/192) | 26 | 476 | 94.8% (476/502) |
|  | 2.912* | 97 | 95 | 67.7% (97/192) | 25 | 477 | 95.0% (477/502) |
|  | 2.931 | 96 | 96 | 67.7% (96/192) | 24 | 478 | 95.2% (478/502) |
| CPS-LFI and | 2.822 | 93 | 99 | 68.8% (93/192) | 34 | 468 | 93.2% (468/502) |
| OPS-ELISA | 2.839 | 93 | 99 | 68.8% (93/192) | 32 | 470 | 93.6% (470/502) |
|  | 2.841 | 93 | 99 | 68.8% (93/192) | 31 | 471 | 93.8% (471/502) |
|  | 2.844 | 93 | 99 | 68.8% (93/192) | 30 | 472 | 94.0% (472/502) |
|  | 2.861 | 93 | 99 | 68.8% (93/192) | 29 | 473 | 94.2% (473/502) |
|  | 2.865 | 93 | 99 | 68.8% (93/192) | 28 | 474 | 94.4% (474/502) |
|  | 3.065 | 81 | 111 | 64.1% (81/192) | 27 | 475 | 94.6% (475/502) |
|  | 3.077 | 79 | 113 | 63.0% (79/192) | 26 | 476 | 94.8% (476/502) |
|  | 3.100* | 79 | 113 | 63.0% (79/192) | 25 | 477 | 95.0% (477/502) |
|  | 3.189 | 76 | 116 | 62.0% (76/192) | 24 | 478 | 95.2% (478/502) |

***** The lowest OD cut-offs that gave a specificity of the ELISA at 95%.
