# Supplementary material for: Evaluation of antigen-detecting and antibody-detecting diagnostic test combinations for diagnosing melioidosis
Source: PLoS Negl Trop Dis. 2021 Nov 2;15(11):e0009840. doi: 10.1371/journal.pntd.0009840 (PMC8562799; doi:10.1371/journal.pntd.0009840)
Supplement: S4 Table — (DOCX) [file pntd.0009840.s004.docx]

**S4 Table. Diagnostic test results in different groups of control patients**

| Characteristics | CPS-LFI  (% negativity) | P-value | Hcp1-ELISA  (% negativity) | P-value | CPS-LFI +  Hcp1-ELISA  (% negativity) | P-value | OPS-ELISA  (% negativity) | P-value | CPS-LFI +  OPS-ELISA  (% negativity) | P-value |
| --- | --- | --- | --- | --- | --- | --- | --- | --- | --- | --- |
| Groups |  |  |  |  |  |  |  |  |  |  |
| Blood culture positive for  *E. coli* | 98.8% (167/169) | 0.86 | 97% (164/169) | 0.003 | 95.7% (162/169) | 0.05 | 95.3% (167/169) | 0.04 | 94.7% (160/169) | 0.18 |
| Blood culture positive for  *K. pneumoniae* | 100% (39/39) |  | 94.9% (37/39) |  | 97.4% (38/39) |  | 97.4% (38/39) |  | 97.4% (38/39) |  |
| Blood culture positive for  *S. aureus* | 100% (20/20) |  | 90.0% (18/20) |  | 100% (20/20) |  | 90.0% (18/20) |  | 90.0% (18/20) |  |
| PCR positive for malaria | 98.0% (148/151) |  | 90.1% (136/151) |  | 90.7% (137/151) |  | 91.4% (138/151) |  | 92.7% (140/151) |  |
| PCR positive for dengue | 98.4% (122/123) |  | 99.2% (122/123) |  | 97.6% (120/123) |  | 99.2% (122/123) |  | 98.4% (121/123) |  |
| Duration of symptoms |  |  |  |  |  |  |  |  |  |  |
| 1-2 days | 98.4% (188/191) | 0.42* | 96.3% (184/191) | 0.14* | 96.9% (185/191) | 0.10* | 97.9% (187/191) | 0.05* | 96.3% (184/191) | 0.39* |
| 3-6 days | 98.4% (249/253) |  | 95.3% (241/253) |  | 94.9 % (240/253) |  | 94.1% (238/253) |  | 94.5% (239/253) |  |
| 7-13 days | 100% (42/42) |  | 88.1% (37/42) |  | 88.1% (37/42) |  | 88.1% (37/42) |  | 92.9% (39/42) |  |
| ≥14 days | 100% (16/16) |  | 93.8% (15/16) |  | 93.8% (15/16) |  | 93.8% (15/16) |  | 93.8% (15/16) |  |
| Modified SOFA score |  |  |  |  |  |  |  |  |  |  |
| 0-1 | 98.7% (73/74) | 0.44* | 98.7% (73/74) | 0.08* | 97.3% (72/74) | 0.08* | 98.7% (73/74) | 0.02* | 97.3% (72/74) | 0.02* |
| 2-3 | 99% (102/103) |  | 96.1% (99/103) |  | 96.1% (99/103) |  | 98.1% (101/103) |  | 99% (102/103) |  |
| 4-5 | 99.4% (156/157) |  | 94.3% (148/157) |  | 96.2% (151/157) |  | 93.6% (147/157) |  | 94.3% (148/157) |  |
| ≥6 | 97.6% (164/168) |  | 93.5% (157/168) |  | 92.3% (155/168) |  | 92.9% (156/168) |  | 92.3% (155/168) |  |
| 28-day mortality |  |  |  |  |  |  |  |  |  |  |
| Died | 2.0% (1/51) | 0.72 | 5.9% (3/51) | 0.76 | 7.8% (4/51) | 0.32 | 3.9% (2/51) | 0.71 | 3.9% (2/51) | 0.71 |
| Survived | 1.3% (6/451) |  | 4.9% (22/451) |  | 4.7% (21/451) |  | 5.1% (23/451) |  | 5.1% (23/451) |  |

Hcp1-ELISA used the OD cut-off value at a specificity of 95% (OD 2.758). Hcp1-ELISA in the CPS-LFI and Hcp1-ELISA combination used the OD cut-off value at a specificity of 95% (OD 2.912). OPS-ELISA used the OD cut-off value at a specificity of 95% (OD 2.839). OPS-ELISA in the CPS-LFI and OPS-ELISA combination used the OD cut-off value at a specificity of 95% (OD 3.100). * P-value for trend
