# Supplementary material for: Evaluation of antigen-detecting and antibody-detecting diagnostic test combinations for diagnosing melioidosis
Source: PLoS Negl Trop Dis. 2021 Nov 2;15(11):e0009840. doi: 10.1371/journal.pntd.0009840 (PMC8562799; doi:10.1371/journal.pntd.0009840)
Supplement: S5 Table — (DOCX) [file pntd.0009840.s005.docx]

**S5 Table. OD values of ELISA in different groups of control patients**

| Characteristics | Hcp1-ELISA  (Median OD, IQR) | P-value | OPS-ELISA  (Median OD, IQR) | P-value |
| --- | --- | --- | --- | --- |
| Groups |  |  |  |  |
| blood culture positive for  *E. coli* | 0.279 (0.108-0.704) | 0.001 | 0.601 (0.280-1.382) | 0.002 |
| blood culture positive for  *K. pneumoniae* | 0.390 (0.148-1.350) |  | 0.729 (0.306-1.658) |  |
| blood culture positive for  *S. aureus* | 0.188 (0.115-0.325) |  | 0.590 (0.356-1.886) |  |
| PCR positive for malaria | 0.432 (0.162-1.137) |  | 0.774 (0.327-1.397) |  |
| PCR positive for dengue | 0.192 (0.104-0.570) |  | 0.399 (0.149-1.007) |  |
