# Supplementary material for: Evaluation of antigen-detecting and antibody-detecting diagnostic test combinations for diagnosing melioidosis
Source: PLoS Negl Trop Dis. 2021 Nov 2;15(11):e0009840. doi: 10.1371/journal.pntd.0009840 (PMC8562799; doi:10.1371/journal.pntd.0009840)
Supplement: Attachment — Submitted filename: PNTD review June 2021.docx [file pntd.0009840.s006.docx]

In this submission by Amornchai et al, they evaluated combinations of a lateral flow immunoassay (LFI) detecting B. pseudomallei capsular polysaccharide (CPS) and enzyme-linked immunosorbent assays (ELISA) detecting antibodies against hemolysin co-regulated protein (Hcp1) or O-polysaccharide (OPS) for diagnosing melioidosis. ELISAs were performed using the CPS and Hcp1 antigens while the data for the LFI was obtained in a previous study. Using data on sensitivity and specificity obtained from this (ELISA) and the previous (LFI) studies, assessment of diagnostic tests in combination was considered positive if either test was positive. Using a relatively high cut-off OD for the Hcp1-ELISA, the combination of the CPS-LFI and Hcp1-ELISA had a sensitivity of 67.7% and a specificity of 95.0%. Sensitivity of the combination was higher than that of CPS-LFI alone or Hcp1-ELISA alone. A similar phenomenon was also observed for the combination of CPS-LFI and OPS-ELISA. The authors went on to conclude that the combined antigen-antibody diagnostic tests increased the sensitivity of melioidosis diagnosis over individual tests while preserving high specificity.

My main concern with this submission is the lack of a strong justification to assess the LFI-ELISA combination as a potential diagnostic practice in endemic areas for melioidosis. What are the implications, time-wise and cost-wise, for a diagnostic laboratory to have to perform both tests using 2 different platforms?

As noted from an extensive literature search as well as the information provided within the Introduction section of the manuscript, many studies (including numerous by this team of authors) have already been conducted on the AMD-LFI, Hcp1 and OPS-based ELISAs, ICT-Hcp1 on different cohorts of melioidosis patients and healthy donor controls. Each of these publications acknowledges that all these various tests cannot achieve the sensitivity and specificity of the gold standard culture-based diagnosis. Furthermore, there are many compounding factors that influence the level of sensitivity and specificity of these tests, such as when the blood samples were obtained, number of days that patients have been showing symptoms before being seen by the physician, the read-out of both tests (visual for the LFI and quantitative for the ELISA), antigen preparation, antibody dilutions used etc.

Specific comments:

1. Sensitivity and specificity data from this study as well as previous literature indicates good values for both ELISAs. As noted above, apart from the 98% specificity obtained using the CPS-LFI, there should be a stronger rationale to justify this study.
2. Pg 9: There doesn’t seem to be any technical (negative) controls for the ELISA.
3. There appears to be no discussion in terms of co-infection (B. pseudomallei and a second bacterial infection) – how does the LFI-ELISA combination perform?
4. Whilst the authors note that the OD cutoff was determined based on achieving 95% specificity, the utilized cutoffs are rather high and this is most likely affecting the sensitivity. The ELISA cutoff was more than twice used in the study by Pumpuang et al (2018 PNTD).
5. Pumpuang et al 2018 also noted that the Hcp1 ELISA test was more promising for the detection of acute melioidosis but the data in this submission indicates that sensitivity of the Hcp1 ELISA is highest in the second week of symptoms?
6. The LFI-ELISA combination appears to be useful for more accurate diagnosis only in patients showing symptoms of < 7 days. What is the true situation in an endemic are like Thailand i.e. how likely is an individual to see the physician with the first 2-3 days of showing symptoms?
7. The data presented in Table 1: I believe the data for the LFI are from the previous publication and therefore has to be acknowledged as such. As the LFI screens were performed 2-3 years before the ELISA (my assumption based on the time line of the 2 papers), are the results truly comparable based on the age of the samples?
8. Table 1: The blood samples were obtained within 24 hours of hospital admission but how does this compare to the timeline of symptoms (Figures 3 and 4)?
9. The McNemar test was used to compare the sensitivity and specificity data between the different tests. Was further analysis e.g. Kapp analysis performed to determine the agreement between different tests?
10. Supplementary table – I suggest that the ELISA data is also represented as negative or positive rather than provide numbers.
