# Supplementary material for: Evaluation of antigen-detecting and antibody-detecting diagnostic test combinations for diagnosing melioidosis
Source: PLoS Negl Trop Dis. 2021 Nov 2;15(11):e0009840. doi: 10.1371/journal.pntd.0009840 (PMC8562799; doi:10.1371/journal.pntd.0009840)
Supplement: Attachment — Submitted filename: Response to reviewers.docx [file pntd.0009840.s007.docx]

**Re: PNTD-D-21-00622: “Evaluation of antigen-detecting and antibody-detecting diagnostic test combinations for diagnosing melioidosis”**

Dear Dr Husain Poonawala and Prof. Anna Ralph

Thank you very much for the reviewers’ suggestions and the opportunity to revise our manuscript. We have addressed the suggestions accordingly, as follows.

**Reviewer #1:**

This paper uses a subset of a cohort of patients from a sepsis study in NE Thailand to evaluate a combination of antibody and antigen detection in serum for the diagnosis of melioidosis and demonstrates the superior sensitivity of this combined approach compared with individual tests, although this was still only 67.7% even when compared with the imperfect gold standard of culture. Whilst I agree that this is an important development, the fact that it will still miss many patients with melioidosis should be given greater prominence.

***Answer: The following sentence has been added into the discussion as suggested, “While preserving high specificity of the combination, the sensitivity of the combination was only 67.7%. This means that this combination may still miss a moderate proportion of melioidosis patients.”***

It is implicit that the tests were only conducted on serum, although the CPS antigen detection test can also be used on other samples, such as pus, sputum and urine, and in such samples it may have higher sensitivity, but this is not made explicit until the discussion, so this should be made clear earlier in the manuscript.

***Answer: The following sentence has been added into the introduction as suggested, “It is possible that using a CPS-LFI in both serum and all non-blood specimens collected systematically from melioidosis suspected patients could increase the sensitivity of the combination further.”***

There are a few minor typos that need correcting:

1. Line 93: change 'accounts' to 'account'.

2. Line 123: change 'placing' to 'place'.

3. Line 131: change 'detects' to 'detect'.

4. Line 432: change 'features' to 'has'.

***Answer: the typos have been corrected as suggested.***

**Reviewer #2:**

Some experiments lack appropriate controls. Some of the methodology is written very briefly and requires the reader to look for the referenced protocol. Not all journals are easily accessible especially for those in countries where melioidosis is endemic. The sample size is adequate but I question the comparison that was done on the same samples but 3 years (?) apart with no statistical analysis between tests.

***Answer: Details of the controls for the experiment has been added in the method section as follows, “Pooled melioidosis sera (5 patients with culture-confirmed melioidosis) and pooled healthy sera (5 healthy Thai donors) were used as positive and negative sera controls for ELISAs.” Details of the ELISA procedures have been added in the method section as follows, “For ELISA procedures, the microtiter plates were prepared by using optimized antigen concentrations of 2.5 µg/mL for Hcp1 and 1 µg/mL for OPS (50 µl per well for coating) and plates were incubated at 4°C for overnight. The plates were then washed 4 times with wash buffer using a Hydrospeed microplate washer followed by blocking with 5% skim milk in PBS at 37°C for 2 hours and were further washed as described above. Fifty microliters of serum diluted (1:2000) was added to wells and incubated at room temperature for 30 min. After washing, 50 µl of 1:2000 horseradish peroxidase-conjugated rabbit antihuman IgG (Dako, Denmark) was added and incubated at room temperature for 30 min. After last washing, 50 µl of TMB substrate solution (Invitrogen, USA) was added and incubated at room temperature for 15 min. The reaction was then stopped with 50 µl of 1N HCl and followed by the optical density (OD) measurement. All ELISAs were conducted in duplicate, and the absorbance values (optical density [OD]) were determined at a wavelength of 450 nm using a microtiter plate reader [19].” The limitation that the test was performed 3 years apart was added as follows, “All ELISAs were conducted on specimens frozen at the time of patient enrollment [21], not on freeze-thaw specimens from any previous studies.” and “The sera were tested for CPS-LFI in 2017 [15] and tested for ELISAs in 2020. We acknowledge that different durations of specimen storage could affect the performance of diagnostic tests.”.***

The limitations are identified but taken together and including other limitations not specifically mentioned in this submission makes it somewhat difficult to appreciate the novelty and applicability of the data in a public health setting, particularly in melioidosis endemic countries.

***Answer: More limitations have been added for clarity. We are thankful for the reviewer’s comment. We do hope that the main finding of our work that “the combination of antigen and antibody-detection diagnostic tests significantly improved the sensitivity of melioidosis diagnosis, compared to when the tests were used individually, while also maintaining high specificity.” would be helpful and evaluated further by researchers in melioidosis-endemic countries. Also, up to our knowledge, this is the first work that evaluate AMD-LFI, Hcp1 and OPS-based ELISAs as a combination diagnostic test.***

In this submission by Amornchai et al, they evaluated combinations of a lateral flow immunoassay (LFI) detecting *B. pseudomallei* capsular polysaccharide (CPS) and enzyme-linked immunosorbent assays (ELISA) detecting antibodies against hemolysin co-regulated protein (Hcp1) or O-polysaccharide (OPS) for diagnosing melioidosis. ELISAs were performed using the CPS and Hcp1 antigens while the data for the LFI was obtained in a previous study. Using data on sensitivity and specificity obtained from this (ELISA) and the previous (LFI) studies, assessment of diagnostic tests in combination was considered positive if either test was positive. Using a relatively high cut-off OD for the Hcp1-ELISA, the combination of the CPS-LFI and Hcp1-ELISA had a sensitivity of 67.7% and a specificity of 95.0%. Sensitivity of the combination was higher than that of CPS-LFI alone or Hcp1-ELISA alone. A similar phenomenon was also observed for the combination of CPS-LFI and OPS-ELISA. The authors went on to conclude that the combined antigen-antibody diagnostic tests increased the sensitivity of melioidosis diagnosis over individual tests while preserving high specificity.

My main concern with this submission is the lack of a strong justification to assess the LFI-ELISA combination as a potential diagnostic practice in endemic areas for melioidosis. What are the implications, time-wise and cost-wise, for a diagnostic laboratory to have to perform both tests using 2 different platforms?

***Answer: The rationale for different platforms has been added for clarity as follows, “The CPS-LFI was selected as an antigen-detecting diagnostic test under evaluation because it is in a point-of-care test that can be used to diagnose melioidosis in 15 minutes, has high specificity, and appears promising for resource-limited melioidosis-endemic settings [14-18]. Hcp1 and OPS-ELISA was selected as an antibody-detecting diagnostic test under evaluation because of its promising sensitivity and specificity [19-20].” and “We also acknowledge that ELISAs under evaluation are not point-of-care tests as we need to define optimal cutoffs for ELISA when used in combination.” Time-wise and cost-wise evaluations were not included in the study.***

As noted from an extensive literature search as well as the information provided within the Introduction section of the manuscript, many studies (including numerous by this team of authors) have already been conducted on the AMD-LFI, Hcp1 and OPS-based ELISAs, ICT-Hcp1 on different cohorts of melioidosis patients and healthy donor controls. Each of these publications acknowledges that all these various tests cannot achieve the sensitivity and specificity of the gold standard culture-based diagnosis. Furthermore, there are many compounding factors that influence

***Answer: We are thankful to the reviewer’s comment, which is also highlighted by the reviewer 1. The limitation has been added for clarity, “While preserving high specificity of the combination, the sensitivity of the combination was only 67.7%. This means that this combination may still miss a moderate proportion of melioidosis patients.” Also, up to our knowledge, this is the first work that evaluate AMD-LFI, Hcp1 and OPS-based ELISAs as a combination diagnostic test.***

**Specific comments from reviewers:**

1. Sensitivity and specificity data from this study as well as previous literature indicates good values for both ELISAs. As noted above, apart from the 98% specificity obtained using the CPS-LFI, there should be a stronger rationale to justify this study.

***Answer: The rationale of selecting CPS-LFI has been added for clarity as follows, “The CPS-LFI was selected as an antigen-detecting diagnostic test under evaluation because it is in a point-of-care test that can be used to diagnose melioidosis in 15 minutes, has high specificity, and appears promising for resource-limited melioidosis-endemic settings [14-18].”***

2. Page 9: There doesn’t seem to be any technical (negative) controls for the ELISA.

***Answer: Details of controls for the ELISAs have been added in the ELISA procedures (Line 201-203 in manuscript) as follows, “Pooled melioidosis sera (5 patients with culture-confirmed melioidosis) and pooled healthy sera (5 healthy Thai donors) were used as positive and negative sera controls for ELISAs.”***

3. There appears to be no discussion in terms of co-infection (*B. pseudomallei* and a second bacterial infection) – how does the LFI-ELISA combination perform?

***Answer. We have not shown the results specific to case patients with co-infection because only two patients (1%) in melioidosis case patients had co-infection. One patient was co-infected with K. pneumoniae and another one was co-infected with Streptococcus group B. They had negative results for all three tests (CPS-LFI, Hcp1- ELISA and OPS-ELISA). However, this could be a spurious finding due to a very limited sample size of two.***

4. Whilst the authors note that the OD cutoff was determined based on achieving 95% specificity, the utilized cutoffs are rather high and this is most likely affecting the sensitivity. The ELISA cutoff was more than twice used in the study by Pumpuang et al (2018 PNTD).

***Answer:*** ***Thank you for your specific comment. You’re correct. To preserve high specificity by using high OD cutoff affects the sensitivity. We have provided the rationale in the introduction as follow, “Misuse of diagnostic tests with low specificity, such as the IHA can lead to misdiagnoses, and thus, a lack of public health responses against melioidosis in Thailand [12].” We think that any diagnosis test that had low specificity should not be used to diagnose melioidosis in endemic-areas. Therefore, the high cutoff point with specificity of 95% was selected to use in this study.***

5. Pumpuang et al 2018 also noted that the Hcp1 ELISA test was more promising for the detection of acute melioidosis but the data in this submission indicates that sensitivity of the Hcp1 ELISA is highest in the second week of symptoms?

***Answer: Different patient characteristics and different OD cutoff for Hcp1-ELISA were used in Pumpuang et al and this study. This is because we would like to preserve high specificity of the combination diagnostic tests and those influenced to the sensitivity of Hcp1-ELISA. Patient samples used in this study were obtained from a cohort of patients presenting with community-acquired infection and sepsis in northeast Thailand (Ubon-sepsis) whose blood was sampled within 24 hours of study hospital admission, while patient samples used in Pumpuang et al’s study were obtained from multiple populations and different time points during illness (e.g. samples were obtained from case patients at a median of 5 days after hospital admission). Our data suggest that the Hcp1-ELISA has higher sensitivity to detect melioidosis patients who present at the hospital and have a long history of symptoms (e.g. >14 days) than those who present at the hospital and have a short history of symptoms (e.g. 1-2 days).***

6. The LFI-ELISA combination appears to be useful for more accurate diagnosis only in patients showing symptoms of < 7 days. What is the true situation in an endemic are like Thailand i.e. how likely is an individual to see the physician with the first 2-3 days of showing symptoms?

***Answer: As shown in the demographics of the case patients “Of 192 case patients, 44, 64, 44 and 40 reported having symptoms prior to admission for 1-2 days, 3-6 days, 7-13 days and ≥14 days, respectively.”, melioidosis patients presenting at the hospital in an endemic area like Thailand have a wide range of clinical characteristics. Therefore, about 23% (44/192) of the melioidosis case patients within the first 2-3 days of symptoms come and see the physician at the study hospital.***

7. The data presented in Table 1: I believe the data for the LFI are from the previous publication and therefore has to be acknowledged as such. As the LFI screens were performed 2-3 years before the ELISA (my assumption based on the time line of the 2 papers), are the results truly comparable based on the age of the samples?

***Answer: Yes, you are correct. Details of the data of the LFI and the limitation has been added as follows, “Results from a study using this CPS-LFI alone were previously published [******15******], and used in this study.” and* “*The sera were tested for CPS-LFI in 2017 [******15******] and tested for ELISAs in 2020. We acknowledge that different durations of specimen storage could affect the performance of diagnostic tests. We also acknowledge that ELISAs under evaluation are not point-of-care tests as we need to define optimal cutoffs for ELISA when used in combination.”***

8. Table 1: The blood samples were obtained within 24 hours of hospital admission but how does this compare to the timeline of symptoms (Figures 3 and 4)?

***Answer: The symptoms were the duration of symptoms prior to admission as noted in the result section, “Of 192 case patients, 44, 64, 44 and 40 reported having symptoms prior to admission for 1-2 days, 3-6 days, 7-13 days and ≥14 days, respectively.” The term “prior to admission” has been added to the title of Figure 3 and 4 for clarity.***

9. The McNemar test was used to compare the sensitivity and specificity data between the different tests. Was further analysis e.g. Kappa analysis performed to determine the agreement between different tests?

***Answer: The McNemar test is the standard and recommended diagnostic test to compare the sensitivities and specificities for the evaluation of two diagnostic tests. (STARD guideline*** [***https://bmjopen.bmj.com/content/bmjopen/6/11/e012799.full.pdf***](https://bmjopen.bmj.com/content/bmjopen/6/11/e012799.full.pdf) ***and Stat Methods Med Res. 2017 Feb;26(1):142-154). Kappa analysis can provide the agreement between tests and is not within the scope of the study.***

10. Supplementary table – I suggest that the ELISA data is also represented as negative or positive rather than provide numbers.

***Answer: The supplementary Table I has been revised as suggested.***

**Reviewer#3**

This manuscript describes the use of 3 immunological based assays to diagnose melioidosis, a fatal tropical disease, in patients in Northeast Thailand. Their findings have suggested that the assays would be more effective if they were used in combinations: CPS-LFI and Hcp1-ELISA, or CPS-LFI and OPS-ELISA. These combinations improved the sensitivity, while the specificity of the combinations remained at 95% or more. However, the adjusted OD cut-off values of ELISA need to be used to achieve the acceptable sensitivity and specificity when the assays were used in a combination. This is a good finding since these 3 assays have been validated in multiple studies earlier. However, the manuscript is not well written, and the calculations may be inaccurate. Here are some comments:

Major comments:

1. It was not mentioned how the sensitivity or the specificity was calculated. Based on their findings, it is unlikely that the authors used the false positive value to calculate the sensitivity, and the false negative value to calculate the specificity. Please note that the sensitivity of an assay is the proportion of the diseased people correctly identified, while the specificity of same assay is the proportion of non-diseased people correctly identified.

***Answer***: ***We are thankful to the reviewer’s comment. Sensitivity was estimated as the proportion of the diseases people correctly identified while the specificity was estimated as the proportion of non-diseased people correctly identified as noted. We added following sentences for clarity. “The sensitivity of diagnostic tests was defined as the proportion of melioidosis case patients who had positive test results. The specificity of diagnostic tests was defined as the proportion of control patients who had negative test results.” Supplementary Table 1 and 2 also display the proportions clearly.***

Did the authors observe the false positive or false negative results at all? If they did, these values need to be included in formulas above. The authors will need to mention in the manuscript if there were false positive or false negative results.

***Answer: Yes, we did observe case melioidosis patients who had negative diagnostic test results and control patients who had positive diagnostic test results. Definitions of the diagnostic tests have been added for clarity. Supplementary Table 1 and 2 have also been modified to display the numbers of case patients with positive and negative tests results, and the number of control patients with positive and negative results clearly.***

2. It has been known that *B. pseudomallei* has diverse LPS types. Is OPS-ELISA able to detect immunoglobulin responses in sera from patients who were infected by B. pseudomallei strains with other LPS/O-antigen types? Has this been tested? Since the atypical LPS types (B, or rough) have been detected in B. pseudomallei strains from Thailand, targeting only the LPS type A by this assay may limit the efficacy of this assay. The authors will need to discuss this limitation.

***Answer: We are thankful to the reviewer’s comment. The following sentences have been added as suggested, “Our study did not evaluate LPS type of B. pseudomallei isolated from the case patients. B. pseudomallei LPS can be categorized to typical type A, atypical types B1 and B2, and rough type to represent the difference of OPS. The OPS-ELISA used in the previous and this study was prepared from B. pseudomallei LPS type A [19, 25], and may have limited sensitivity among patients infected with B. pseudomallei with atypical or rough LPS type.”***

3. Are both Hcp1-ELISA and OPS-ELISA detecting igG, IgM, or total IgG? It is worth mentioning it, otherwise the readers have to check with ref. 19 and 20 for more details. Since both assays have been compared in ref. 19, and the Hcp1-ELISA was the best one, why did the authors compare both assays again in this study? Were the patients' sera used in this study from the same cohort used in ref. 19?

***Answer: Both Hcp1-ELISA and OPS ELISA detects total IgG. We added this detail in introduction as follows, “Additionally, enzyme-linked immunosorbent assays (ELISA) that detect specific IgG antibodies against hemolysin co-regulated protein (Hcp1) or Type A O-polysaccharide (OPS) have been developed.” We included both Hcp1-ELISA and OPS-ELISA in order to evaluate them with different cutoffs and in a different population. Details of the data of the LFI has been added as follows, “Results from a study using this CPS-LFI alone were previously published [******15******], and used in this study.” and “All ELISAs were conducted on specimens frozen at the time of patient enrollment [21], not on freeze-thaw specimens from any previous studies.”***

4) It was not clear if the authors tested all these 3 assays at the same time in this study, or only used the results from previous studies for statistical analysis? Please clarify this since the authors cited ref. 15 and 19 in most parts of their methods.

***Answer: Details of the data of the LFI and the limitation has been added as follows, “Results from a study using this CPS-LFI alone were previously published [******15******], and used in this study.” and* “*The sera were tested for CPS-LFI in 2017 [******15******] and tested for ELISAs in 2020. We acknowledge that different durations of specimen storage could affect the performance of diagnostic tests. We also acknowledge that ELISAs under evaluation are not point-of-care tests as we need to define optimal cutoffs for ELISA when used in combination.”***

Other comments:

1. In the last statement of the Author Summary, the authors should suggest whether the combination, CPS-LFI and Hcp1-ELISA, or CPS-LFI and OPS-ELISA, is further evaluated for the point-of-care diagnosis of melioidosis rather than saying in general that "using combinations of antigen- and antibody-detection should be further developed and evaluated".

***Answer: We would like to remain using the term “combinations of antigen- and antibody-detection”. This is because we evaluated the concept of combinations of antigen- and antibody-detection for diagnosis of melioidosis, while preserving high specificity of the combination diagnostic test.***

2. Line: 126-128: Did the authors mean the sensitivity of PCR was limited in detecting B. pseudomallei in clinical specimens?

***Answer: Yes, we meant that the sensitivity of PCR is limited in detecting B. pseudomallei in clinical specimens. In the real clinical settings, PCR has a sensitivity of 61% in Thailand (Chantratia et al, 2007) and PCR on blood samples had a sensitivity of 56% from bacteremic melioidosis patients in Australia (Menmann et al. 2006). Additional references had been added in the introduction.***

***All contributing authors have reviewed and concurred with the revised manuscript.***

***Yours,***

***Premjit Amornchai***
