# Supplementary material for: Correction: Evaluation of antigen-detecting and antibody-detecting diagnostic test combinations for diagnosing melioidosis
Source: PLoS Negl Trop Dis. 2022 Jun 2;16(6):e0010095. doi: 10.1371/journal.pntd.0010095 (PMC9162502; doi:10.1371/journal.pntd.0010095)
Supplement: S2 Table — This table includes supplementary data. (DOCX) [file pntd.0010095.s001.docx]

**S2 Table. Sensitivity and specificity of a combination between CPS-LFI and Hcp1-ELISA and a combination of CPS-LFI and OPS-ELISA using different OD cut-off values**

| Assay | OD cut-off  (of ELISA) | Cases (N = 192) | | Sensitivity | Controls (N = 502) | | Specificity |
| --- | --- | --- | --- | --- | --- | --- | --- |
|  |  | No. of cases with positive results | No. of cases negative results | (No. of cases with positive results/N) | No. of controls with positive results | No. of controls with negative results | (No. of controls with negative results/N) |
| CPS-LFI and | 2.721 | 137 | 55 | 71.4% (137/192) | 33 | 469 | 93.4% (469/502) |
| Hcp1-ELISA | 2.758 | 136 | 56 | 70.8% (136/192) | 32 | 470 | 93.6% (470/502) |
|  | 2.797 | 133 | 59 | 69.3% (133/192) | 30 | 472 | 94.0% (472/502) |
|  | 2.824 | 132 | 60 | 68.8% (132/192) | 28 | 474 | 94.4% (474/502) |
|  | 2.832 | 132 | 60 | 68.8% (132/192) | 27 | 475 | 94.6% (475/502) |
|  | 2.896 | 130 | 62 | 67.7% (130/192) | 26 | 476 | 94.8% (476/502) |
|  | 2.912* | 130 | 62 | 67.7% (130/192) | 25 | 477 | 95.0% (477/502) |
|  | 2.931 | 130 | 62 | 67.7% (130/192) | 24 | 478 | 95.2% (478/502) |
| CPS-LFI and | 2.822 | 132 | 60 | 68.8% (132/192) | 34 | 468 | 93.2% (468/502) |
| OPS-ELISA | 2.839 | 132 | 60 | 68.8% (132/192) | 32 | 470 | 93.6% (470/502) |
|  | 2.841 | 132 | 60 | 68.8% (132/192) | 31 | 471 | 93.8% (471/502) |
|  | 2.844 | 132 | 60 | 68.8% (132/192) | 30 | 472 | 94.0% (472/502) |
|  | 2.861 | 132 | 60 | 68.8% (132/192) | 29 | 473 | 94.2% (473/502) |
|  | 2.865 | 132 | 60 | 68.8% (132/192) | 28 | 474 | 94.4% (474/502) |
|  | 3.065 | 123 | 69 | 64.1% (123/192) | 27 | 475 | 94.6% (475/502) |
|  | 3.077 | 121 | 71 | 63.0% (121/192) | 26 | 476 | 94.8% (476/502) |
|  | 3.100* | 121 | 71 | 63.0% (121/192) | 25 | 477 | 95.0% (477/502) |
|  | 3.189 | 119 | 73 | 62.0% (119/192) | 24 | 478 | 95.2% (478/502) |

***** The lowest OD cut-offs that gave a specificity of the ELISA at 95%.
