# Supplementary material for: Correction: Evaluation of antigen-detecting and antibody-detecting diagnostic test combinations for diagnosing melioidosis
Source: PLoS Negl Trop Dis. 2022 Jun 2;16(6):e0010095. doi: 10.1371/journal.pntd.0010095 (PMC9162502; doi:10.1371/journal.pntd.0010095)
Supplement: S3 Table — This table includes supplementary data. (DOCX) [file pntd.0010095.s002.docx]

**S3 Table. Diagnostic test results in different groups of melioidosis case patients**

| Characteristics | CPS-LFI  (% positivity) | P-value | Hcp1-ELISA  (% positivity) | P-value | CPS-LFI +  Hcp1-ELISA  (% positivity) | P-value | OPS-ELISA  (% positivity) | P-value | CPS-LFI + OPS-ELISA  (% positivity) | P-value |
| --- | --- | --- | --- | --- | --- | --- | --- | --- | --- | --- |
| Duration of symptoms |  |  |  |  |  |  |  |  |  |  |
| 1-2 days | 36.4% (16/44) | 0.004* | 45.5% (20/44) | <0.001* | 65.9% (29/44) | 0.15* | 43.2% (19/44) | 0.04* | 61.4% (27/44) | 0.15* |
| 3-6 days | 45.3% (29/64) |  | 37.5% (24/64) |  | 65.6% (42/64) |  | 37.5% (24/64) |  | 73.4% (47/64) |  |
| 7-13 days | 18.2% (8/44) |  | 63.6% (28/44) |  | 61.4% (27/44) |  | 54.6% (24/44) |  | 52.3% (23/44) |  |
| ≥14 days | 17.5% (7/40) |  | 77.5% (31/40) |  | 80.0% (32/40) |  | 65.0% (26/40) |  | 60.0% (24/40) |  |
| Modified SOFA score |  |  |  |  |  |  |  |  |  |  |
| 0-1 | 7.1% (3/42) | <0.001* | 66.7% (28/42) | 0.03* | 69.1% (29/42) | 0.62* | 57.1% (24/42) | 0.41* | 52.4% (22/42) | 0.006* |
| 2-3 | 18.2% (4/22) |  | 59.1% (13/22) |  | 63.6% (14/22) |  | 40.9% (9/22) |  | 50.0% (11/22) |  |
| 4-5 | 24.4% (10/41) |  | 51.2% (21/41) |  | 58.5% (24/41) |  | 46.3% (19/41) |  | 56.1% (23/41) |  |
| ≥6 | 49.4% (43/87) |  | 47.1% (41/87) |  | 72.4% (63/87) |  | 47.1% (41/87) |  | 74.7% (65/87) |  |
| Blood culture positive for *B. pseudomallei* |  |  |  |  |  |  |  |  |  |  |
| Yes | 38% (57/150) | <0.001 | 49.3% (74/150) | 0.02 | 68% (102/150) | 0.87 | 44.7% (67/150) | 0.05 | 64.7% (97/150) | 0.37 |
| No | 7.1% (3/42) |  | 69.1% (29/42) |  | 66.7% (28/42) |  | 61.9% (26/42) |  | 57.1% (24/42) |  |
| 28-day mortality |  |  |  |  |  |  |  |  |  |  |
| Died | 56.6% (56/99) | <0.001 | 45.5% (45/99) | 0.02 | 64.7% (64/99) | 0.35 | 44.4% (44/99) | 0.25 | 64.7% (64/99) | 0.63 |
| Survived | 18.3% (17/93) |  | 62.4% (58/93) |  | 71.0% (66/93) |  | 52.7% (49/93) |  | 61.3% (57/93) |  |

Hcp1-ELISA used the OD cut-off value at a specificity of 95% (OD 2.758). Hcp1-ELISA in the CPS-LFI and Hcp1-ELISA combination used the OD cut-off value at a specificity of 95% (OD 2.912). OPS-ELISA used the OD cut-off value at a specificity of 95% (OD 2.839). OPS-ELISA in the CPS-LFI and OPS-ELISA combination used the OD cut-off value at a specificity of 95% (OD 3.100). * P-value for trend
